# Studying Alzheimer’s‐Related Neuronal Dysfunction Using a New Tool: Characterizing a Neurodegenerative Animal Model with Increased Neuronal‐specific miR34

**DOI:** 10.1002/alz.092993

**Published:** 2025-01-03

**Authors:** India A Pursell, Raul Freitas, Laura Wegrowski, Gabrielle Blahusiak, Natasha B Barahona, Elizabeth Engler‐Chiurazzi

**Affiliations:** ^1^ Tulane University, New Orleans, LA USA

## Abstract

**Background:**

Alzheimer’s Disease (AD) is a prevalent age‐related neurodegenerative condition leading to dementia, yet factors regulating its polygenomic etiology and progression remain elusive. MicroRNAs (miRNAs), small RNA molecules regulating protein expression, play a role in neurodegeneration. MicroRNA‐34a (miR‐34a) is a crucial regulator of numerous genes associated with neurodegenerative disorders, protein aggregation and synaptic transmission genes. However, the lack of effective tools for in vivo study impedes comprehensive research in this area. We developed a global miR‐34a overexpressing mouse line in which cognitive deficits, altered amyloid and tau protein processing, and synaptic reorganization were noted.

**Method:**

We generated a CaMKIIα driven, excitatory neuron‐specific Tet‐inducible miR‐34a overexpressing mouse model. Male and female miR‐34a± mice aged 28‐46 weeks were treated with water or Doxycycline to induce miR‐34a overexpression for up to 90 days cognitive and neurobiological consequences were assessed. In this pilot study, we assessed the expression of predicted miR‐34a target genes and associated proteins (SIRT1, NMDAR2B). A subset of mice underwent sequencing analysis in one hemisphere to identify novel miR‐34a targets.

**Result:**

At 30 days of miR‐34a overexpression, we observed trends towards reduced levels of miR‐34a target genes (NMDAR2B, SHANK3) in the hippocampus of mice. However, these findings were non‐significant, likely attributed to the limited power or insufficient exposure duration. With 90 days of exposure, mice exhibited subtle behavior changes, characterized by a decreased percent of spontaneous alternations; no differences observed at 30 days. Distinct nervous system cell populations were isolated for miRNA analysis, hinting at region‐specific changes. Whole brain hemisphere bulk RNA sequencing of Doxy‐treated animals revealed upregulation of the genes Gh and Prl and downregulation of Avp and Drd1 compared to control.

**Conclusion:**

The inducible global miR‐34a overexpression mouse model exhibited modest dementia‐like characteristics and warrants further characterization. Further interrogation of contributions from other neural or peripheral cell types in the AD phenotype induced by global miR‐34a overexpression. The cell‐specific nature of our model provides valuable insights into miR‐34a’s role in AD pathology. These findings contribute to understanding the complex, polygenomic mechanisms underlying neurodegeneration in AD and may reveal novel miR‐focused therapeutic targets.

